# Characterization of *Pseudomonas* spp. and Associated Proteolytic Properties in Raw Milk Stored at Low Temperatures

**DOI:** 10.3389/fmicb.2017.02158

**Published:** 2017-11-08

**Authors:** Lu Meng, Yangdong Zhang, Huimin Liu, Shengguo Zhao, Jiaqi Wang, Nan Zheng

**Affiliations:** ^1^Ministry of Agriculture Laboratory of Quality and Safety Risk Assessment for Dairy Products, Institute of Animal Science, Chinese Academy of Agricultural Sciences, Beijing, China; ^2^Ministry of Agriculture Milk and Dairy Product Inspection Center, Beijing, China

**Keywords:** milk, *Pseudomonas* spp., proteolytic activities, *apr*X gene, spoilage

## Abstract

Milk spoilage is caused by the presence of proteolytic enzymes produced by *Pseudomonas* spp. during storage at low temperatures. The aim of this study was to identify *Pseudomonas* spp. in raw milk and investigate their associated proteolytic properties at low temperatures. Raw milk samples (*n* = 87) were collected from 87 bulk tanks in Shaanxi Province in China. *Pseudomonas* spp. were identified using *Pseudomonas* specific 16S, universal *16S rRNA* sequencing, and *rpo*B gene sequencing. The proteolytic properties of *Pseudomonas* spp. were examined using milk agar, quantitative trinitrobenzenesulfonic acid assay, and by the presence of alkaline metallopeptidase gene (*apr*X). A total 143 isolates from all 87 samples were confirmed as *Pseudomonas*, and were identified as belonging to 14 *Pseudomonas* species. Of these, 40 (28.0%) isolates revealed proteolysis on milk agar at 2°C, 74 (51.8%) at 4°C, 104 (72.7%) at 7°C, and 102 (71.3%) at 10°C. However, proteolytic activity of 45 (31.5%) isolates exceeded 2 μmol of glycine equivalents per mL at 7°C, followed by 43 (30.1%) at 10°C, 18 (12.6%) at 4°C, and 7 (4.9%) at 2°C. The results reveal proteolytic activity of *Pseudomonas* spp. present in milk and their spoilage potential at different temperatures.

## Introduction

Spoilage of milk resulting from the contamination of dairy products with psychrotrophic microorganisms results in significant losses for the food industry and is a particular concern of the dairy industry (Dogan and Boor, 2003). Milk is usually stored at low temperatures for 2 to 5 days prior to heat treatment (De Jonghe et al., 2011; Baur et al., 2015). During storage, the microbiota shifts toward psychrotrophic microorganisms, which can reduce the quality of raw milk (Lafarge et al., 2004; Xin et al., 2017).

*Pseudomonas* has been identified as predominant milk-associated psychrotrophic bacteria, making it one of the most important bacterial groups in the dairy industry (Wiedmann et al., 2000; Marchand et al., 2009a). The most commonly detected *Pseudomonas* species in milk and milk products are *P. fluorescens, P. gessardii, P. fragi*, and *P. lundensis* (Mallet et al., 2012). *Pseudomonas* spp. can grow over a temperature range of 4–42°C, with an optimal growth temperature above 20°C (Chakravarty and Gregory, 2015). They are present in different environments and are frequently linked to food spoilage, especially, that of raw milk (Quigley et al., 2013; Chakravarty and Gregory, 2015). *Pseudomonas* can outgrow other bacteria at low temperatures, accounting for at least 50% of all bacteria in milk (Munsch-Alatossava and Alatossava, 2006; Fricker et al., 2011; von Neubeck et al., 2015). The growth of the *Pseudomonas* is often associated with the production of extracellular enzymes (e.g., peptidases and lipases).

Peptidases secreted by *Pseudomonas* during cold storage are heat-stable extracellular peptidases that can retain their activity after pasteurization or ultrahigh temperature (UHT) treatment (Marchand et al., 2009b; Glück et al., 2016). The residual enzyme activities can cause coagulation and degradation of milk and dairy products over time. They mainly belong to the class of metallopeptidase (EC 3.4.24.) (Dufour et al., 2008; Scatamburlo et al., 2015; Caldera et al., 2016). The most important peptidase, a heat-resistant peptidase alkaline metallopeptidase (AprX), belongs to the serralysin family, and has been characterized in several strains of *Pseudomonas* spp. It is responsible for the spoilage of milk with activity on casein (Dufour et al., 2008). The spoilage ability of *Pseudomonas* spp. varies dramatically depending on the strain and growth conditions (Chabeaud et al., 2001; Nicodeme et al., 2005). Many studies have quantified the proteolytic activity of *Pseudomonas* spp. within the temperature range 4–7°C (Kumaresan et al., 2007; Baur et al., 2015; Caldera et al., 2016). However, the assay conditions used in these studies did not simulate conditions during transport and at dairy plants (Machado et al., 2017). Furthermore, a low temperature (1–4°C) is recommended for the long-term storage of raw milk before further processing (De Jonghe et al., 2011).

The aims of the present work were (i) to isolate and identify *Pseudomonas* spp. from the raw cows’ milk samples; and (ii) to investigate the associated proteolytic properties stored at low temperatures (2, 4, 7, and 10°C, respectively).

## Materials and Methods

### Sampling of Raw Milk and the Identification of *Pseudomonas* spp.

Raw milk samples (*n* = 87; 25 mL each) were collected directly from 87 bulk tanks of 87 farms in Shaanxi Province in China in spring (average daily temperature > 20°C), when *Pseudomonas* spp. have high prevalence. The 87 farms were randomly chosen and were mainly located in Xi’an, Xianyang, Weinan, and Tongchuan, where the main milk producing areas are found (herd size ≤ 300, milking frequency 2–3 times per day, no clinical mastitis cow). All samples were transferred to sterile plastic bottles (Corning Inc., Corning, NY, United States), stored at 4°C, and transported to the laboratory within 4 h.

For the isolation and detection of *Pseudomonas* spp., all raw milk samples were first processed as described by Scatamburlo et al. (2015). Briefly, all raw milk samples were diluted 10-fold in 0.85% NaCl (wt/vol) and homogenized. Aliquots (1 mL) of selected dilutions were placed onto *Pseudomonas* agar (Oxoid Ltd., Basingstoke, United Kingdom) to selectively isolate *Pseudomonas* spp. The plates were incubated at 25°C for 48 h. Colonies (5–8 per plate) were then streaked onto new *Pseudomonas* agar plates (Oxoid), and incubated at 25°C for 48 h.

Single colonies were chosen from *Pseudomonas* agar and incubated in LB broth (Beijing Land Bridge Technology Co., Ltd., Beijing, China) overnight at 25°C. DNA was extracted using the InstaGene Matrix DNA extraction kit (Bio-Rad Laboratories, Hercules, CA, United States) according the manufacturer’s instructions. *Pseudomonas* spp. were then identified by PCR (Bio-Rad Laboratories). The primers were synthesized by GeneCreate Biological Engineering Co., Ltd. (Wuhan, China). A negative control (a sample without genomic DNA) and a positive control (DNA of *P. fluorescens* CICC 21620; China Center of Industrial Culture Collection, Beijing, China) were included in all PCR assays.

*Pseudomonas* spp. were initially identified by PCR amplification of the 16S DNA region. The following primers were used: PA-GS-F (5′-GACGGGTGAGTAATGCCTA-3′) and PA-GS-R (5′-CACTGGTGTTCCTTCCTATA-3′) (Spilker et al., 2004). The PCR reaction conditions were as described in Scatamburlo et al. (2015). Amplicons (618 bp) were considered indicative of *Pseudomonas* spp.

Isolates identified as *Pseudomonas* spp. (*n* = 143) were further analyzed by amplification of the *16S rRNA* fragment and *rpo*B sequences using universal primers 27F (5′-AGAGTTTGATCCTGGCTCAG-3′) and 1492R (5′-CTACGGCTACCTTGTTACGA-3′), and PSF (5′-AGTTCATGGACCAGAACAACC-3′) and PTR (5′-CCTTGACGGTGAACTCGTTTC-3′) (Lane et al., 1985; Sajben et al., 2011). The amplification programs were performed according to Sajben et al. (2011) and Caldera et al. (2016).

Finally, the obtained amplicons were sequenced at Beijing Genomics Institute (Beijing, China). Sequence data of the isolated *Pseudomonas* species were analyzed using the Basic Local Alignments Search Tool (BLAST) program available from the National Center for Biotechnology Information (NCBI)^1^. During universal *16S rRNA* fragment and *rpo*B sequence analysis, the cut-off level for pairwise comparisons was 99%.

### Plate Assays of Proteolytic Activity

*Pseudomonas* single colonies were streaked onto *Pseudomonas* agar (Oxoid Ltd.) supplemented with penicillin (100,000 IU/L, Dr. Ehrenstorfer GmbH, Augsburg, Germany), pimaricin (0.01 g/L, Dr. Ehrenstorfer GmbH), and UHT milk [10%, vol/vol, Modern Farming (Group) Co., Ltd., Hebei, China] (henceforth referred to as “milk agar”) (Scatamburlo et al., 2015). The plates were incubated at 2, 4, 7, and 10°C for 5 days. The temperature of 25°C was set as a control. The plates were monitored daily. Proteolytic halos in the inoculated areas were indicative of proteolytic activity (Scatamburlo et al., 2015). The plate assays were performed twice.

### Proteolytic Activity Quantification

To induce peptidases production at 2, 4, 7, and 10°C, *Pseudomonas* spp. isolates were first incubated in 5 mL of UHT milk [Modern Farming (Group) Co., Ltd.] for 5 days. Then the proteolytic activity was determined according to the protocol described by Caldera et al. (2016) to quantify the native proteolytic activity at different temperatures.

The trinitrobenzenesulfonic acid (TNBS) method was used to monitor the presence of free α-amino groups, indicators of protein hydrolysis (Polychroniadou, 1988; Marchand et al., 2009a). The TNBS reagent (Sigma–Aldrich, Taufkirchen, Germany) was reacted with the released α-amino groups at pH 9.2 in the dark for 100 min. The intensity of the yellow-orange color of the reaction products was measured by absorption values at 420 nm (Varioskan^TM^ Flash Multimode Reader, Thermo Fisher Scientific, Waltham, MA, United States). Native proteolytic enzyme levels in raw milk were determined from the absorption ratios. Three independent replicate measurements were performed. *Pseudomonas* spp. isolates were considered peptidase active if the measured absorption exceeded 2 μmol glycine equivalents per mL. Standard curve was generated using glycine (Sigma–Aldrich).

### Identification of *apr*X Gene

The amplification of the *apr*X gene from *Pseudomonas* spp. isolates was performed using SM2F (5′-AAATCGATAGCTTCAGCCAT-3′) and SM3R (5′-TTGAGGTTGATCTTCTGGTT-3′) primers according to Caldera et al. (2016). Amplicons of ca. 850 bp were considered typical for *apr*X.

## Results

### Molecular Classification and Identification of *Pseudomonas* spp.

Genus-specific PCR of 16S DNA fragments (618 bp) was used for preliminary characterization of their assignment at the *Pseudomonas* genus level. In total 143 *Pseudomonas* isolates were confirmed. In Supplementary Table S1 identifications are given for each isolate based on universal *16S rRNA* and *rpo*B gene sequencing analyses. The 14 different *Pseudomonas* species (**Table 1**) were mainly based on the *rpo*B gene sequence data, since universal *16S rRNA* gene sequences are less discriminative (Caldera et al., 2016).

**Table 1:** Identification of *Pseudomonas* spp. in raw milk sampled from 87 farms.

Group	Species	No. of isolates
*Pseudomonas fluorescens* group	*Pseudomonas azotoformans*	6
	*Pseudomonas brenneri*	2
	*Pseudomonas cedrina*	5
	*Pseudomonas fluorescens*	61
	*Pseudomonas gessardii*	5
	*Pseudomonas poae*	4
	*Pseudomonas proteolytica*	1
*Pseudomonas chlororaphis* group	*Pseudomonas fragi*	37
	*Pseudomonas lundensis*	2
*Pseudomonas putida* group	*Pseudomonas putida*	2
Unknown group	*Pseudomonas baetica*	2
	*Pseudomonas deceptionensis*	1
	*Pseudomonas lurida*	2
	*Pseudomonas psychrophila*	13
Total		143

In the current study, the 14 *Pseudomonas* species are divided into 4 different groups, according to Anzai et al. (2000), namely, *P. fluorescens* group, *P. chlororaphis* group, *P. putida* group, and unknown group. The amount of *P. fluorescens* group (*n* = 84) was dominant, followed by *P. chlororaphis* group (*n* = 39) (**Table 1**).

### Proteolytic Activity of the Identified *Pseudomonas* spp. Isolates

The detailed phenotypic results of *Pseudomonas* isolates were shown in **Table 2** and Supplementary Table S1. We evaluated the proteolytic activity of the isolates at all tested temperatures. Extracellular peptidase activity on milk agar was determined by the appearance of halo in the inoculated area. Following a 5 days incubation on milk agar, extracellular peptidase activity was detected in 74.1% (106/143) of the isolates at 25°C, around 72% of the isolates at 7°C (104/143) and 10°C (102/143), 51.8% (74/143) at 4°C and 28.0% (40/143) at 2°C. No directly correlations were observed between *Pseudomonas* groups and the degree of proteolysis (Supplementary Table S1).

**Table 2:** *Pseudomonas* spp. isolates^1^ with observable proteolytic activity^2^ at different temperatures.

Day	Incubation temperature (°C)				
	2	4	7	10	25
1	2	5	8	16	54
2	6	12	26	33	15
3	12	27	39	31	12
4	4	11	14	13	17
5	16	19	17	9	8
Total	40	74	104	102	106

*^1^The total number of *Pseudomonas* spp. isolates obtained from raw milk was 143.*

*^2^As determined during growth on milk agar. The number of new isolates exhibiting extracellular proteolytic activity on a given day of incubation on milk agar is shown.*

### Quantification of the Proteolytic Activity of *Pseudomonas* spp. Isolates

**FIGURE 1:**
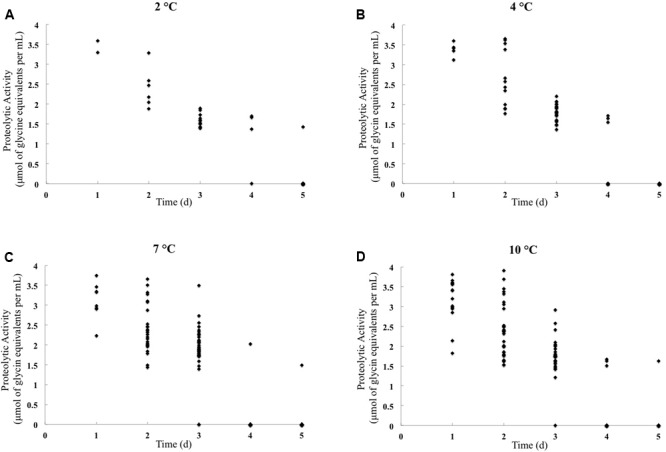
Proteolytic activity quantification of *Pseudomonas* isolates obtained from raw cow milk samples stored for 5 days at 2°C **(A)**, 4°C **(B)**, 7°C **(C)**, and 10°C **(D)**. The *X*-axis represents the storage day when the isolates displayed extracellular peptidase activity on milk agar, and the *Y*-axis represents proteolytic activity (μmol of glycine equivalents per mL). Species names and isolate numbers are not indicated.

The peptidase activity was quantified using UHT milk, and the results are shown in **Figure 1** and Supplementary Table S1. In the standard curve, relating glycine concentration to absorbance in the TNBS assay, the *R*^2^ value was greater than 0.99 (data not shown). Strains were considered proteolytic active when the measured proteolytic activity exceeded 2 μmol of glycine equivalents per mL.

The highest percentage of proteolytic activity isolates was obtained at 7°C (31.5%, *n* = 45) and 10°C (30.1%, *n* = 43). And 4.9% (7/143) isolates were proteolytic active at 2°C, and 12.6% (18/143) at 4°C. Using the TNBS method, peptidase activity was detected in 17.5% (7/40) of the isolates exhibiting extracellular peptidase activity on milk agar at 2°C and in 24.3% (18/74) of the isolates that were active at 4°C. At 7 and 10°C, the number of peptidase activity isolates was much larger than that at 2 and 4°C, reached 43.3% (45/104) and 42.2% (43/102), respectively (**Table 2**).

### Analysis of the *apr*X Gene

We amplified the *apr*X gene from all isolates using PCR. An amplicon of the expected size (±850 bp) was obtained from reactions with genomic DNA of 133/143 isolates. Moreover, all the isolates, which displayed extracellular peptidase activity on milk agar, could detect the presence of *apr*X gene.

## Discussion

After molecular confirmation at the genus level, the 143 isolates were assigned at species level. In general, we found a large diversity of *Pseudomonas* species. The frequencies of the *Pseudomonas* spp. confirm the findings of Jayarao and Wang (1999), who reported 98 isolates belonging to nine *Pseudomonas* species. Moreover, the *Pseudomonas* isolates were found to be randomly distributed among the different farms. These results demonstrate the ubiquitousness of *Pseudomonas* spp. in dairy farms, highlighting their relevance as natural contaminants (Scatamburlo et al., 2015).

PCR sequencing confirmed that 42.7% (*n* = 61) of the isolates were *P. fluorescens*, the dominant *Pseudomonas* specie. Our results are in agreement with those of Chambers (2003), who reported that *P. fluorescens* was the dominant *Pseudomonas* spp. in milk and those of Dogan and Boor (2003), who identified 51% of *Pseudomonas* isolates as *P. fluorescens* in fluid milk products and dairy processing plants using API20 NE. *P. putida* is another species of *Pseudomonas* found in milk (Senel and Gürsoy, 2014); however, we found only 2 *P. putida* isolates in the *P. putida* group in the current study (**Table 1**). *P. psychrophila* was the third most common strain identified here. By contrast, Vithanage et al. (2014) found only a few *P. psychrophila* isolates among psychrotrophic bacteria in raw milk samples from a commercial UHT milk processor. Although *Pseudomonas* spp. are ubiquitous in raw milk, our findings indicate that *Pseudomonas* spp. in milk differ considerably across studies. These differences could stem from regional and environmental differences.

All the isolates showed growth over a wide range of temperatures (2–25°C), although many studies have demonstrated that the growth of *Pseudomonas* isolates is temperature-dependent (Munsch-Alatossava and Alatossava, 2006; Caldera et al., 2016). By contrast, Munsch-Alatossava and Alatossava (2006) reported that *Pseudomonas* strains isolated from farms do not grow at 4 or 7°C. This could be because of differences in the growth characterization of the isolates.

The results showed that most isolates produced peptidase at 25°C, followed by 7, 10, 4, and 2°C. Similarly, peptidase production by *P. fluorescens* was reported previously to be highest at 22°C, followed by 7 and 32°C (Wang and Jayarao, 2001). Therefore, our results confirm those of other studies and demonstrate the spoilage capability of *Pseudomonas* spp. during storage and transport at low temperatures. In addition, we demonstrated that a number of *Pseudomonas* spp. isolates exhibit extracellular peptidase activity at low temperature after a short period of time, stressing the importance of controlling contamination during the early steps of milk storage.

Proteolytic activity was then confirmed by TNBS quantitative analysis and expressed as glycine equivalents per mL. The number of peptidase active isolates increased with temperature, most likely reflecting the fact that proteolytic activity is temperature regulated (Woods et al., 2001; McCarthy et al., 2004). Taken together, the extracellular peptidase activity and proteolytic activity data showed that the isolates could produce peptidases at different temperatures. This is a pertinent observation as it confirms the ability of *Pseudomonas* species to produce proteolytic enzymes under psychrotrophic conditions. More isolates produced peptidases with an increase in temperature; however, different peptidases were active at different temperatures (Supplementary Table S1).

According to current laws and standards in many countries, raw milk must be stored below 7°C (FDA, 2013; Scatamburlo et al., 2015; Zheng, 2015). Many studies have found that *Pseudomonas* spp. can produce peptidases under storage temperatures. Caldera et al. (2016) reported that 46.8% (15/32) of *Pseudomonas* isolates from milk and dairy products after incubation at 5°C for 5 days were peptidase active using a quantitative assay. Moreover, when Kumaresan et al. (2007) assessed the native proteolytic activity of psychrotrophs at 2, 4, and 7°C using the Kjeldahl method, they found that the storage of raw milk at these temperatures resulted in peptidase production by *Pseudomonas* spp. In this study, we confirmed these findings by showing that a number of *Pseudomonas* isolates could produce peptidases and exhibit proteolytic activity under different storage temperatures. Proteolytic activity was lower at 2°C than at 4°C, 7°C, or 10°C, as assessed by the TNBS method. However, we also identified many *Pseudomonas* isolates that acquired higher proteolytic activity after storage at 2°C than at 4°C, 7°C, and 10°C, indicating that spoilage ability is strain-dependent.

In some countries, the majority of raw milk is not processed after milking until delivery to a dairy, which might last up to 3 or 4 days. Additional storage before processing may also take place in the dairy (Fricker et al., 2011; von Neubeck et al., 2015). Therefore, 5 days was chosen to study the peptidase activities of the isolates in this study. Kumaresan et al. (2007) demonstrated that proteolytic activity increased with storage time. Stoeckel et al. (2016) also found a linear correlation between onset of product defects and enzyme activity in UHT milk samples. Our results showed that peptidases could be produced by a number of *Pseudomonas* isolates between 2°C and 10°C and that proteolytic activity increased with storage time. The peptidases secreted by *Pseudomonas* spp. in raw milk stored at low temperature are stable during heat treatment, which might explain the persistent proteolytic activity observed during storage of processed milk (Marchand et al., 2009a; Glück et al., 2016). Therefore, in order to prolong the storage time of milk products, raw milk should be preferentially stored at low temperature and processed within 48 h after milking to prevent the release of thermostable spoilage peptidases by *Pseudomonas* spp.

There was a good correlation between extracellular peptidase activity on milk agar and the presence of the *apr*X as determined by PCR, confirming the role of AprX in milk degradation as reported previously (Dufour et al., 2008; Caldera et al., 2016). However, some *apr*X-positive isolates do not show proteolytic activity at any storage temperature, indicating that AprX might be inhibited during milk protein degradation. In the genus *Pseudomonas*, the *apr*X gene is usually involved in nutrient utilization; the product of this gene degrades extracellular proteins, and therefore *Pseudomonas* spp. are usually associated with the spoilage of milk and dairy products (Dufour et al., 2008; Marchand et al., 2009b; Zhang et al., 2009). The production of this enzyme highlights the controlling of *Pseudomonas* spp. contamination in raw milk and dairy products.

## Conclusion

This study revealed a diverse *Pseudomonas* spp. population in raw milk. In the dairy, milk is usually stored at temperatures below 7°C before processing. This temperature does not prevent the growth and proteolytic activity of *Pseudomonas* spp. in raw milk. Although all the *Pseudomonas* isolates could grow between 2°C and 10°C, proteolytic activity decreased when the milk storage temperature was reduced from 10°C to 2°C. There was no relation between specific *Pseudomonas* spp. and proteolytic activity. Low storage temperature and short periods before processing (within 48 h) could reduce peptidase production of *Pseudomonas* spp., but milking hygiene should also be properly controlled. It is necessary to acquire more information on *Pseudomonas* spp. with proteolytic activity and to develop sensitive and efficient tools to monitor for the presence of peptidases in raw milk.

## Author Contributions

LM performed the major experiments and wrote the manuscript. YZ did many work in experiments and helped in writing. HL helped in the research and writing. SZ and JW gave the help in research plans. NZ is the corresponding author.

## Conflict of Interest Statement

The authors declare that the research was conducted in the absence of any commercial or financial relationships that could be construed as a potential conflict of interest.

## Footnotes

1 https://blast.ncbi.nlm.nih.gov/Blast.cgi
